# Indirect Correspondence-Based Robust Extrinsic Calibration of LiDAR and Camera

**DOI:** 10.3390/s16060933

**Published:** 2016-06-22

**Authors:** Sungdae Sim, Juil Sock, Kiho Kwak

**Affiliations:** Agency for Defense Development, P.O.Box 35, Yuseong, Daejeon 34186, Korea; sungdae.sim@gmail.com (S.S.); juil.sock@gmail.com (J.S.)

**Keywords:** extrinsic calibration, indirect correspondence, loop-closing constraint, joint calibration

## Abstract

LiDAR and cameras have been broadly utilized in computer vision and autonomous vehicle applications. However, in order to convert data between the local coordinate systems, we must estimate the rigid body transformation between the sensors. In this paper, we propose a robust extrinsic calibration algorithm that can be implemented easily and has small calibration error. The extrinsic calibration parameters are estimated by minimizing the distance between corresponding features projected onto the image plane. The features are edge and centerline features on a v-shaped calibration target. The proposed algorithm contributes two ways to improve the calibration accuracy. First, we use different weights to distance between a point and a line feature according to the correspondence accuracy of the features. Second, we apply a penalizing function to exclude the influence of outliers in the calibration datasets. Additionally, based on our robust calibration approach for a single LiDAR-camera pair, we introduce a joint calibration that estimates the extrinsic parameters of multiple sensors at once by minimizing one objective function with loop closing constraints. We conduct several experiments to evaluate the performance of our extrinsic calibration algorithm. The experimental results show that our calibration method has better performance than the other approaches.

## 1. Introduction

In recent years, an increasing amount of interest and research effort has been put toward autonomous vehicles from both commercial and military sectors. For safer and more robust navigation, autonomous vehicle should utilize all sensors mounted on the vehicle to perceive and understand the scene around. For instance, the vehicle should be able to detect objects, classify the environment and analyze the condition of the road surface. Although a vision sensor provides rich information, it also has weaknesses, such as a narrow field of view and poor behavior in rapid illumination changes. LiDAR overcomes such drawbacks of the vision sensor. Although LiDAR provides sparser data, it is equipped with a wider field of view, highly accurate depth measurement and robustness to environmental change. Perception systems with sensory fusion have shown better results in various literature works [1,2,3].

To obtain accurate sensor measurements, we should estimate their intrinsic and extrinsic parameters. Generally, the sensors should be calibrated in order to convert data between the local coordinate systems of the sensors. Calibration can be divided into two parts: intrinsic calibration, in which the sensors’ intrinsic parameters, such as focal length, skew parameter and image center, are estimated; and extrinsic calibration, in which the rigid body transformation between the sensors is estimated. Specifically, to optimally combine LiDAR and camera measurements, accurate extrinsic calibration of the six degrees of freedom transformation between the sensors is required. In other words, we should know their relative pose with rotation and translation from each other to effectively use the data from both sensors. Extrinsic calibration between a LiDAR and a camera is roughly classified into two categories: direct and indirect correspondence searches.

The extrinsic calibration problem can be solved by directly finding corresponding features in the LiDAR and camera data [4,5]. However, finding such correspondences is difficult for several reasons. First, the LiDAR spot usually cannot be seen directly in the image, since LiDAR typically operates outside the visual spectrum. Although the spot can be seen with special purpose equipment, such as an infrared camera or scope, most LiDARs cannot be controlled, making it impossible to determine which LiDAR point is being observed. Another challenge is that some types of features, such as intensity edges, can only be detected in one sensor modality. Finally, the single line LiDARs that our algorithm calibrates can measure only 1D structure in the imaging plane.

To address this problem, researchers have proposed using constraints derived from lines and depth edges, rather than points. In these previous works, two types of 3D features are commonly used: points lying at depth edges [6,7] and points lying on 3D corners [8,9]. Intensity edges are often used as image-based features. The extrinsic calibration parameters may be obtained by minimizing the distance between corresponding features either projected onto the image plane or in 3D. However, the existing approaches did not provide any ideas; how to manage the weight of each correspondence that is different from each other and how to handle the effect of outliers in datasets. Moreover, most of the existing approaches propose methods for calibrating a pair of sensor; camera to camera or LiDAR to camera.

Recent approaches for extrinsic calibration between cameras and LiDARs have focused on automatic and targetless method [10,11,12]. However, most automatic approaches need high density 3D points and specific environments to find correspondences. Automatic and targetless approaches have difficulty dealing with low density sensor data. For low density data, some approaches use high precision inertial navigation sensors to make high density sensor data for calibration. Due to these drawbacks, manual-aided correspondence detection and using targets are still good approaches, even though automatic and targetless approaches are the main trend.

**Figure 1:**
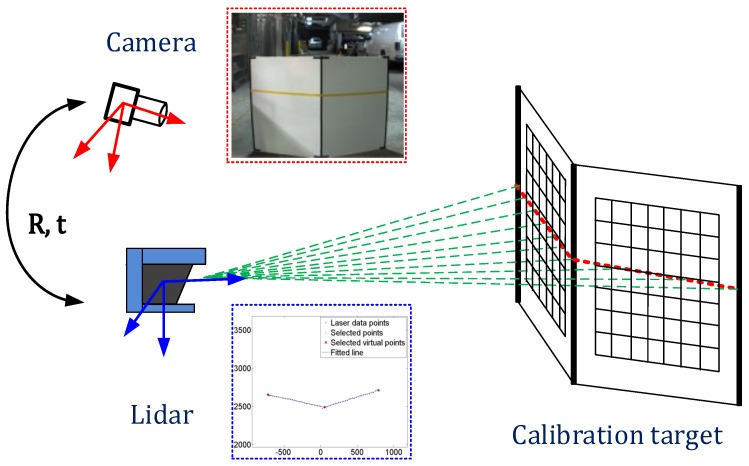
The relative pose between the line scanning LiDAR and the camera is estimated using features extracted from images of a v-shaped target.

In this paper, we address the problem of extrinsic calibration between a 2D/3D LiDAR and a camera. Our extrinsic calibration algorithm follows the existing basic procedure, but provides several advanced techniques to improve the calibration accuracy and a joint calibration method of multiple sensors. We use a v-shape calibration target consisting of two planes with checker patterns, as shown in Figure 1. We collect images of the laser line intersecting the target with different poses and at different ranges. Our 3D features consist of the 3D depth edges on both sides of the target and the 3D corner in the center. Image features are intensity edges extracted from both edges and the centerline of the target. We estimate the extrinsic parameters by minimizing the distance between the 3D features projected on the image plane and the corresponding image edge features. Additionally, based on our calibration techniques for a pair of sensors, we estimate the extrinsic calibration parameters of multiple sensors at once by minimizing one objective function with loop-closing constraints. Since the joint calibration approach use correspondences between images, the checker pattern on the v-shape target is utilized to find them.

The contributions of this paper are three-fold: (1) we introduce new extrinsic calibration approaches to improve the performance of the existing approaches, the weighted and robust calibration algorithms; (2) we propose a joint calibration method of multiple sensors with loop-closing constraints; (3) we provide extensive experimental comparisons with state-of-the-art approaches and statistical analyses of the proposed calibration approach.

## 2. Previous Work

There are several approaches to estimate the extrinsic parameters between a LiDAR and a camera. These approaches can be classified based on whether the correspondences are measured in the same modality or in separate modalities.

The first approach is to directly search for correspondences with a sensor that can observe the LiDAR beam. If the camera is an IR camera, it is possible to detect the LiDAR spots on the image directly. Calibration targets, such as poles or planar boards, can be used to determine which laser point corresponds to an observed image point. For example, if the LiDAR beam hits a pole, we can easily detect that point in the 3D data and also in the image. If we use only a regular camera, the same procedure can be performed using an IR scope by marking the laser position on the target with tape to make it visible in the camera image [4,5].

The second approach is to indirectly estimate the correspondences by finding features separately in each modality. This approach can be also classified by whether the optimization is done in 2D or 3D and by what type of features are used. The approach proposed by Zhang optimizes in 3D [7]. Given the pose of the calibration target in 3D, the extrinsic calibration parameters are estimated by solving a nonlinear optimization problem that minimizes the position error of the LiDAR data with respect to the calibration board in 3D. The constraint that the LiDAR points on the calibration target must be placed on the calibration target at the estimated pose makes this possible. This approach has two problems: (1) it is often a poor guess that leads to a local minimum; and (2) the initial value may not be a valid pose. Vasconcelos addressed these problems in [13]. The author reformulates the extrinsic calibration problem as the one of registering a set of planes and a line in the 3D space. He proposes a minimal solution to the problem in a RANSAC framework and refines it with nonlinear iterative optimization. However, these approaches are hard to calibrate at long range because of the need to detect corners on the checkerboard calibration target. Ramalingam also addressed a minimal solution for the registration of 3D points to 3D planes [14]. It addresses a 3D registration solution, not extrinsic sensor calibration. However, it might be adoptable to extrinsic calibration, which uses 3D plane and 3D point features. For example, it can be adopted to solve the registration between 2D image lines and 3D LiDAR points.

Automatic and targetless approaches are increasingly popular extrinsic calibration methods for cameras and LiDARs. Pandey *et al*. addressed automatic extrinsic calibration by maximizing mutual information between image and 3D LiDAR data [10]. The author uses intensity and reflectivity information to get the cost function. Levinson *et al*. suggest automatic online calibration using edges of the image and the discontinuity of LiDAR points. These approaches have difficulty handling low density LiDAR (such as 2D LiDAR) with respect to camera extrinsic calibration because they need relatively dense 3D points to match dense image data. Scott *et al*. suggest automatic extrinsic calibration between 2D LiDAR and the camera [12]. This approach accumulates LiDAR points using a visual odometry system for a while and matches with image data using the intensities of points. However, this approach needs a highly accurate odometry system to obtain accurate calibration. In addition, all automatic approaches work in structured sites, such as urban environments. They cannot work well in natural environments and can give poor results. Therefore, manual-aided calibration approaches are still good to make a robust operation for various situations.

Li [9] and Wasielewski [8] proposed approaches to optimize in 2D and to use constraints obtained from intensity edges on the image and points lying at depth edges or corners in 3D. The basic ideas of the two approaches are similar in spirit to ours. Li’s approach uses a black triangle panel for the calibration target. Points at depth edges and intensity edges of the calibration target are used for the features. Wasielewski’s approach uses a v-shaped calibration target. The approach uses only a single corresponding feature, the centerline of the calibration target in the image and the maximum curvature point lying on the corner in 3D.

We implemented Li’s and Wasielewski’s algorithms to evaluate the calibration performance and found that the algorithms do not provide sufficiently accurate results. Li’s approach is not accurate due to sparse laser sampling. Although Wasielewski used highly accurate corresponding points lying on corners, he used only one corresponding point per image, and therefore, a large number of images is needed.

Our approach builds on the ideas of Li and Wasielewski to address the problems of each approach. Based on an early version [15,16], this work includes a more thorough analysis, discussions and new experiments for the extrinsic calibration problem of a LiDAR-camera and a LiDAR and multiple cameras. We first extend the previous existing work in four significant ways to achieve superior performance: (1) we use virtual edge points instead of real depth edge points to remove bias at depth edges; (2) we apply weights based on the accuracy of the correspondences; (3) we use a robust approach to handle the effect of outliers in the datasets; and (4) we introduce a joint calibration of a LiDAR and multiple cameras. The proposed method estimates the extrinsic parameters simultaneously by minimizing one objective functions with loop-closing constraints.

## 3. Notation

In order to combine data from a camera and a LiDAR, the extrinsic and intrinsic parameters must be estimated. The transformation is represented by rotation R and translation t between the sensors. A rigid body transformation converts a 3D point $P_{L}$ = $(X_{L}$, $Y_{L}$, $Z_{L})$ in the LiDAR coordinate system to a point $P_{C}$ = $(X_{C}$, $Y_{C}$, $Z_{C})$ in the camera coordinate system:

(1) $$P_{C}=R\cdot P_{L}+t$$

where R is a 3×3 orthonormal rotation matrix and t is a 3×1 translation vector.

A point $P_{C}$ in the camera coordinate system is projected to a pixel p=(u,v) in image coordinates using Equations (2) and (3):

(2) $$\begin{array}{c} \left[\begin{array}{c} x\\ y\\ z \end{array}\right]=\left[\begin{array}{ccc} \alpha_{x} & s & u_{0}\\ 0 & \alpha_{y} & v_{0}\\ 0 & 0 & 1 \end{array}\right]\left[\begin{array}{c} X_{C}\\ Y_{C}\\ Z_{C} \end{array}\right] \end{array}$$

(3) $$\begin{array}{c} \left[\begin{array}{c} u\\ v \end{array}\right]=\left[\begin{array}{c} x/z\\ y/z \end{array}\right] \end{array}$$

where $\alpha_{x}$ and $\alpha_{y}$ are the focal length in pixels, $u_{0}$ and $v_{0}$ are the coordinate of image center in pixels and *s* is the skew parameter [6].

## 4. Extrinsic Calibration Approach

Our extrinsic calibration algorithm consists of three steps: data acquisition, feature extraction and optimization. These steps are described in the following subsections.

### 4.1. Calibration Target and Data Acquisition

**Figure 2:**
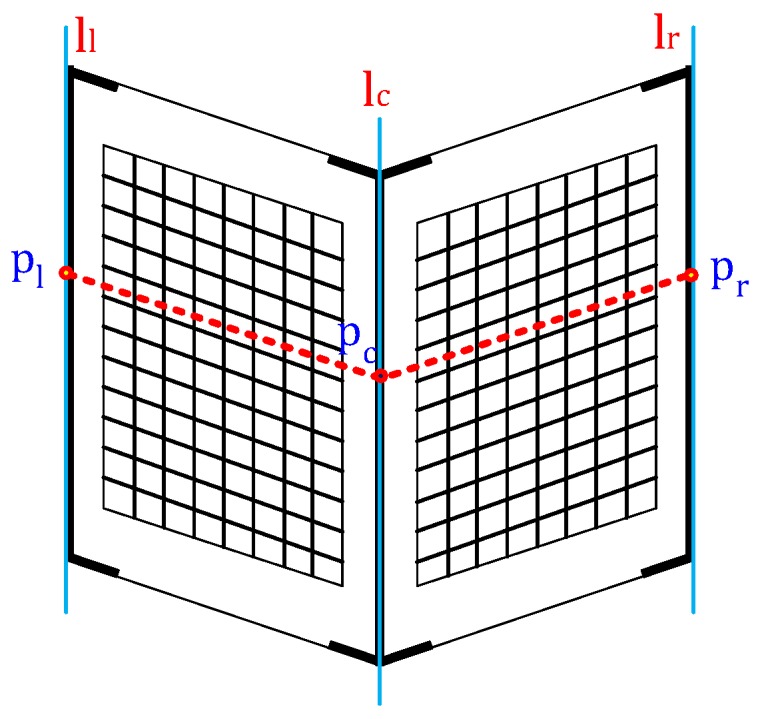
Calibration board description used for the extrinsic calibration: the blue lines (left ($l_{l}$), center ($l_{c}$) and right ($l_{r}$)) describe the line feature that we extracted in the image, and the three red points ($p_{l}$, $p_{c}$, $p_{r}$) are the point features in 3D. The checker pattern on each plane is utilized for the joint calibration.Calibration board description.

The calibration target we designed consists of two planar boards arranged in a v-shape (Figure 2). We marked the centerline and left and right boundaries with black tape to enable those edges to be detected in images and drew checker patterns on each plane to find correspondences between cameras. The checker patterns are only used for the joint calibration of a LiDAR and multiple cameras. We created our target from foamcore boards, which are low cost, light weight and sufficiently planar. Using this calibration target, we obtain a set of images and LiDAR scans with the target in different poses and at different ranges. Both planes on the convex side must be visible to each sensor. The LiDAR scan line must intersect both the left and right sides of the target. The LiDAR and camera must either be synchronized or held stationary during a scan.

### 4.2. Feature Definition and Extraction

**Figure 3:**
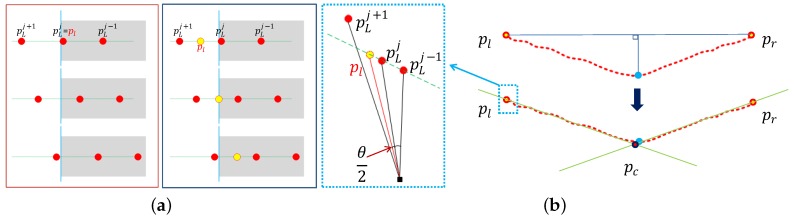
LiDAR feature definition and extraction: We used virtual points (yellow dots) instead of real LiDAR measurements (red dots) for our extrinsic calibration. (**a**) The left boundary point ($p_{L}^{j}$) is never exactly placed on the left edge line of the calibration board. Instead, we select a virtual point ($p_{l}$) between the two consecutive points ($p_{L}^{j}$, $p_{L}^{j+1}$), located at the expected value of the edge line position (yellow dot). In the figure, the gray plane represents the calibration target, and the blue line describes the left edge line of the calibration target; (**b**) The virtual point ($p_{l}$) is selected as a point that is placed on the intersection position of the angular mid-point and the line fitted to the left side LiDAR points. *θ* is the angular resolution of the LiDAR. The center point ($p_{c}$) is selected as the intersection of the left and right fitted lines.

We extract three line features ($l_{l}$, $l_{c}$, $l_{r}$) from each image and three point features ($p_{l}$, $p_{c}$, $p_{r}$) from each LiDAR scan, as shown in Figure 2. The image-based line features correspond to the left and right edges and the centerline of the target. Currently, we manually select the line features because we found it difficult to automatically extract these lines in long-range images. The 3D features extracted from the LiDAR scan include the left and right depth edges and the center point (Figure 3). First, we manually select the border points on the target on the left and right sides ($p_{L}^{j}$, $p_{R}^{j}$). Second, we segment the LiDAR data into two linear segments corresponding to the left and right sides of the target. The segmentation is accomplished using the iterative-end-point-fit (IEPF) algorithm [17]. The IEPF algorithm recursively splits a set of points until a distance-related criterion is satisfied. In our case, the algorithm is stopped after two segments are found. Next, we fit lines to each segment using the total least squares method [18]. Finally, the intersection of those lines is defined as the center point.

Li proposed using edge points as 3D features directly. Li’s method extracts 3D features from a LiDAR and a stereo camera. However, it is impossible to extract 3D features from single cameras. Moreover, since the LiDAR can be sparse, the features may be far from the true edge of the target, especially at long distances. Therefore, we propose using virtual feature points placed at the expected value of the true edge locations, as shown in Figure 3a. If we assume that the true edges are uniformly distributed between the last point ($p_{L}^{j}$) and the next consecutive point ($p_{L}^{j+1}$), the expected value is the mid-point of the two points. The feature ($p_{l}$) is computed by selecting the intersection point of the angular mid-point and the fitted line, as shown in Figure 3b. The feature ($p_{r}$) is computed analogously.

### 4.3. Optimization

The extrinsic parameters are estimated by minimizing the distance between the line features and point features in 2D. Given a set of *N* paired images and LiDAR scans with the target in different poses and at different ranges, we project the point features ($p_{l}$, $p_{c}$, $p_{r}$) in 3D onto the images. In the image plane, we measure the normal distance between each line feature ($l_{l}$, $l_{c}$, $l_{r}$) and corresponding projected point feature (${\tilde{p}}_{l}$, ${\tilde{p}}_{c}$, ${\tilde{p}}_{r}$). We obtain three distance values per image ($d_{l}$, $d_{c}$, $d_{r}$), one each on the left and right borders and from the centerline. The error function E(R,t) is defined as the squared sum of those distances over the *N* image and LiDAR pairs:

(4) $$\begin{array}{c} E(R,\;t)=\sum_{i=1}^{N}\;{\left(d_{l}^{i}\right)}^{2}+{\left(d_{c}^{i}\right)}^{2}+{\left(d_{r}^{i}\right)}^{2}\;\;\;\\ \;d_{k}^{i}=dist({\tilde{p}}_{k}^{i},\;l_{k}^{i}),\;\;k=\{left,\;center,\;right\} \end{array}$$

where the function dist is the minimum distance from point (${\tilde{p}}_{k}$) to line ($l_{k}$). We estimate the extrinsic parameters by minimizing the error function in Equation (4) using the Levenberg–Marquardt (LM) method [19,20].

## 5. Extensions to the Basic Approach

In many systems and applications, the combination of a LiDAR and a camera is broadly utilized because they are complementary to each other. In this section, we introduce new approaches to improve the performance of the basic approach. We observed two characteristics in the basic approach: some points are more reliable than the others, and line features can be noisy due to manual extractions. Therefore, we extended the basic approach in two ways. First we applied different weights to the distance error for the center features versus the edge features because the center feature is more accurate than the others. Second, we incorporated a robust optimization approach to handle the effect of outliers in datasets.

### 5.1. Feature Weighting

Intuitively, the corresponding accuracy of the center feature is higher than the others because the point is selected as the intersection of the left and right fitted lines. We apply more weight to the distance of the center feature. The weighting values are computed by the following steps. First, we measure the average residuals ($e_{l}$, $e_{c}$, $e_{r}$) of each position from Equation (4). The average residuals are computed as $e_{l}=\sum_{i}^{N}\;{\left(d_{l}^{i}\right)}^{2}/N$ where *N* is the number of image and LiDAR scan pairs. The center and right residuals ($e_{c}$ and $e_{r}$) are expressed analogously. Second, we compute the weights ($w_{l}$, $w_{c}$, $w_{r}$) as $w_{l}=1/e_{l}$. Each weight value describes the confidence of the distance measure. We also show that the correspondence of the center feature is more accurate through experiments in Section 8. Once we have determined the weights, we re-estimate the extrinsic parameters once more to balance weights of vertical line features by minimizing the following error function with the LM algorithm:

(5) $$E_{W}\left(R,\;t\right)=\sum_{i=1}^{N}\;w_{l}\cdot {\left(d_{l}^{i}\right)}^{2}+w_{c}\cdot {\left(d_{c}^{i}\right)}^{2}+w_{r}\cdot {\left(d_{r}^{i}\right)}^{2}$$

### 5.2. Robust Optimization

Since we extract the corresponding line and point features manually, noise is likely to be included in image and LiDAR pairs. Small errors can be managed by increasing the size of the dataset, but outliers in the dataset cause the accuracy of the extrinsic calibration to decrease. To eliminate outliers in the dataset, there are several methods, such as RANSAC or MSAC [13]. Since the iterative approaches such as RANSAC and MSAC are suitable for closed-form solutions, it is hard to apply our calibration using nonlinear optimization. We apply a simple approach suggested by Huber to exclude the influence of these outliers [21]. We designed the penalty function as Equation (7). If there exists a large pixel distance error between the selected edge line and the corresponding LiDAR point, the algorithm gives the penalty measured by the Huber penalty function. Otherwise, it returns the distance value. The equation is represented as follows [22]:

(6) $$E_{R}\left(R,\;t\right)=\sum_{i=1}^{n}w_{l}\cdot \phi_{huber}\left(d_{l}^{i}\right)+w_{c}\cdot \phi_{huber}\left(d_{c}^{i}\right)+w_{r}\cdot \phi_{huber}\left(d_{r}^{i}\right)$$

where $\phi_{huber}\left(\cdot \right)$ is the Huber penalty function:

(7) $$\phi_{huber}\left(d\right)=\left\{\begin{array}{cc} d^{2} & \;\text{if}\;\left|d\right|\leq d_{max}\\ d_{max}(2\cdot |d|-d_{max}) & \;\text{if}\;|d|>d_{max} \end{array}\right.$$

Since we use the virtual points instead of the real border points, the maximum distance on the image is bounded as follows:

(8) $$d_{l},\;d_{r}≲d_{max}=f\tan\left(\frac{\theta }{2}\right)$$

where *f* is the focal length of the camera and *θ* is the angular resolution between successive LiDAR points. We use this bound to define $d_{max}$.

## 6. Joint Calibration of a LiDAR and Multiple Cameras

In this section, we introduce a joint calibration method of multiple sensors with loop-closing constraints. There are many autonomous systems and robots consisting of multiple sensors for the purpose and function of the systems. Most existing approaches divide the systems into many pairs of sensors and calibrate each pair at a time. In such cases, one should collect different datasets and use different calibration algorithms. This procedure can often lead to inaccurate calibrations. For example, suppose a sensor 1 is calibrated to a sensor 2, which is calibrated to a sensor 3, and so on in a long chain. If there exists a small amount of error at each step, then the error in the transformation between the sensor 1 and a sensor *n* may become large because the errors along the chain would accumulate. This procedure is accomplished with a sensor configuration consisting of a LiDAR and two cameras. To solve this problem, we assume that there exists an overlapped sensing region between sensors. The extrinsic parameters are estimated by minimizing the total error among the installed sensors.

### 6.1. Loop-Closing Constraints

If a system consists of more than three sensors, it is possible to make a closed loop to connect each sensor. In such a case, a point on an arbitrary coordinate should return to its original position by a transformation T.

Let $T_{i}^{j}$ be the transformation from the *i*-th coordinate to the *j*-th coordinate. The $T_{i}^{j}$ consists of the rotation $R_{i}^{j}$ and translation $t_{i}^{j}$ of the *j*-th coordinate with respect to the *i*-th coordinate. In the homogeneous coordinate, the $T_{i}^{j}$ is given as:

(9) $$\begin{array}{c} T_{i}^{j}=\left(\begin{array}{cc} R_{i}^{j} & t_{i}^{j}\\ 0 & 1 \end{array}\right) \end{array}$$

The transformation from the first sensor to itself in the closed loop is represented as:

(10) $$T_{1}^{1}=T_{n}^{1}T_{n-1}^{n}\cdots T_{2}^{3}T_{1}^{2}=I$$

where $T_{1}^{1}$ should satisfy the identity matrix I.

### 6.2. Total Error Function

In the case of the system consisting of a LiDAR and multiple cameras, if the two cameras form a stereo vision system, the calibration parameters can be estimated by solving an objective function with 3D data as [23]. Otherwise, we should find another method to form an objective function with an equivalent error unit. In Section 5, we already define the error measurement between a LiDAR and a camera to the pixel distance between the line feature on the imagery and the corresponding projected LiDAR point feature. In the case of the joint calibration, we define the error measurement between cameras to the pixel distance, as well.

Once we know the actual size of the target board and the intrinsic parameters of each camera, we can estimate the 3D point $X^{i}$ of the point $x^{i}$ on an image. Let the corresponding image pixels $x_{c1}^{i}$, $x_{c2}^{i}$ be on the image planes of Camera 1 and Camera 2. The 3D points $X_{c1}^{i}$, $X_{c2}^{i}$ corresponding to $x_{c1}^{i}$, $x_{c2}^{i}$ are measured by using the scale factor estimated by the known size target and the intrinsic parameters. The pixel distance can be measured from the difference of a point *x* on the image and the corresponding projected 3D points $\tilde{x}$. The error function $E_{cameras}\left(R,\;t\right)$ between the cameras is defined as the sum of the pixel distances of each camera:

(11) $$E_{Cameras}\left(R,\;t\right)=\sum_{i=1}^{n}∥{\tilde{x}}_{c1}^{i}-x_{c2}^{i}∥$$

where:

$$X_{c1}=\alpha K_{c1}^{-1}x_{c1},\;\;\;\;\;\;{\tilde{x}}_{c1}=K_{c2}\;\left[R_{c1}^{c2}\;\;\;\;t_{c1}^{c2}\right]\;X_{c1}$$

*α* is the scale factor estimated from the known-size target board and $K_{c1,\;c2}$ are the intrinsic parameters of Cameras 1 and 2.

In the case of the sensor system consisting of a LiDAR and two cameras, the extrinsic calibration parameters are estimated by minimizing the total error function with the loop-closing constraints. The total error function is represented as the weighted sum of the error functions of each sensor pair:

(12) $$\begin{array}{cc} \underset{R,\;t}{\text{minimize}} & \;\;\left(w_{1}E_{1}\left(R_{1},\;t_{1}\right)+w_{2}E_{2}\left(R_{2},\;t_{2}\right)+w_{3}E_{3}\left(R_{3},\;t_{3}\right)\right)\\ \text{subject}\;\text{to} & \;\;\;\;\;\;\;\;\;\;\;\;\;R_{3}^{1}R_{2}^{3}R_{1}^{2}=I\\ & \;\;\;\;\;\;\;\;\;\;\;\;\;\left(R_{3}^{1}R_{2}^{3}\right)t_{1}^{2}+R_{3}^{1}t_{2}^{3}+t_{3}^{1}=0 \end{array}$$

where $w_{1,2,3}$ are the normalized weights depending on the quantity of the correspondences of each sensor pair and $\sum_{k=1}^{3}w_{k}=1$.

With Equation (12), we reduce the total error function consisting of two rotations and two translations, while the original function consists of three rotations and three translations. This enforces the optimized calibration results to satisfy the closed-loop constraints.

## 7. Experimental Setup

**Figure 4:**
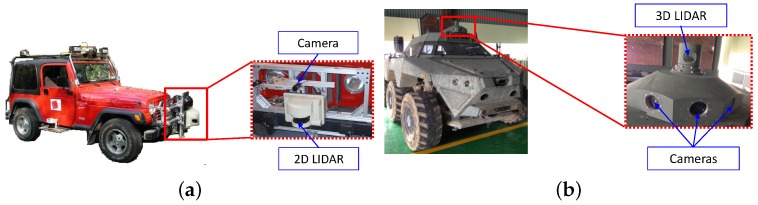
Two test platforms used for the experiments: we used two different sensor configurations for the experiments. (**a**) A single scanning LiDAR and a camera used for the performance evaluation of the proposed weighted and robust calibration approaches; (**b**) 3D LiDAR and cameras used for the joint calibration.

In order to illustrate and evaluate our algorithm, we used two different sensor configurations as shown in Figure 4. The first sensor configuration with a LiDAR and a camera is used to evaluate the performances of the proposed weighted and robust calibration approaches. This sensor pair consists of a SICK LMS-221 LiDAR and a PointGrey Flea2 camera (Figure 4a). The LiDAR have a 180${}^{\circ }$ horizontal field of view, with a line scanning frequency of 37.5 Hz and a 0.5${}^{\circ }$ angular resolution. The cameras have a 60${}^{\circ }$ horizontal field of view, with a frame rate of 15 Hz and a resolution of 1024 by 768 pixels. The LiDAR is attached in front of the vehicle, and the camera lies upside of the LiDAR with a 50-cm distance. The second sensor configuration with a LiDAR and multiple cameras is used to evaluate the performance of the proposed joint calibration. These multiple sensors consist of a Velodyne HDL-32 3D LiDAR and two PointGrey Flea2 cameras (Figure 4b). The LiDARs have a 360${}^{\circ }$ horizontal field of view and a 40${}^{\circ }$ vertical field of view, with a 10-Hz frame rate and a 0.16${}^{\circ }$ angular resolution. The cameras have a 60${}^{\circ }$ horizontal field of view, with a frame rate of 30 Hz and a resolution of 640 by 480 pixels. The data from each sensor are time-stamped to enable synchronization. Sensors are packed in one suit and installed on the roof of the vehicle. The intrinsic parameters of the cameras (e.g., focal length, optical center, pixel aspect ratio and skew) were computed using the MATLAB Camera Calibration Toolbox [24]. During data acquisition, we stop the vehicle at the appropriate place, and we capture sensor data, while we move freely the v-shaped calibration target in front of the sensors. The images used for the experiment were rectified by the intrinsic parameters prior to feature extraction. The relative poses measured by hand roughly are utilized for the initial values of the calibration algorithm. The initial values did not disturb the calibration algorithms to converge to global optimum, as the experimental results show.

**Figure 5:**
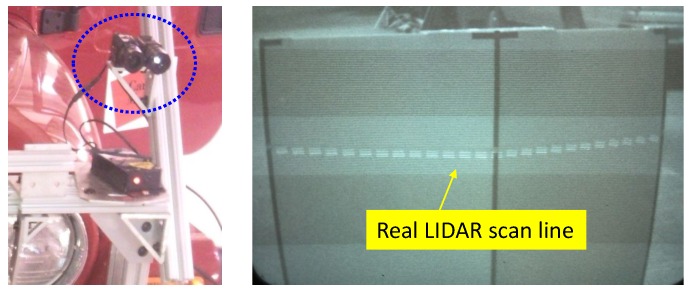
Ground truth generation: we detected the LiDAR scan using an IR camera (blue circle) (**Left**). The detected scan line was visible on a monitor (**Right**).

**Figure 6:**
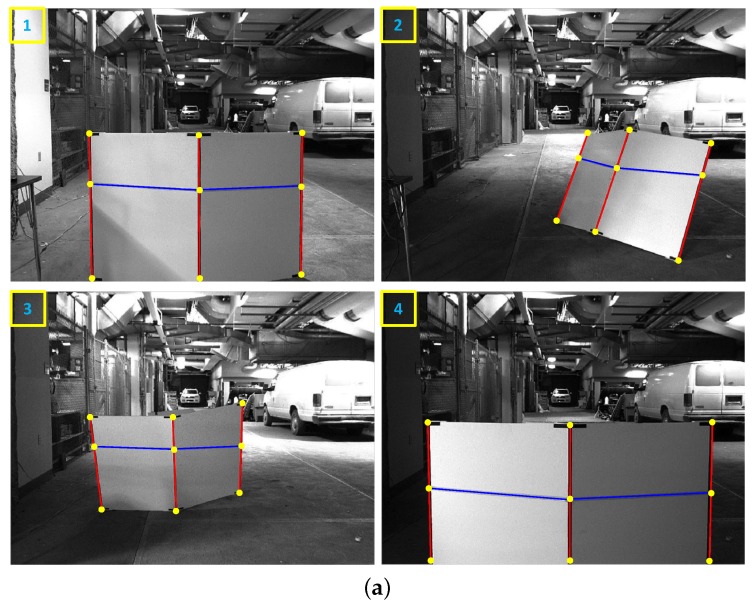

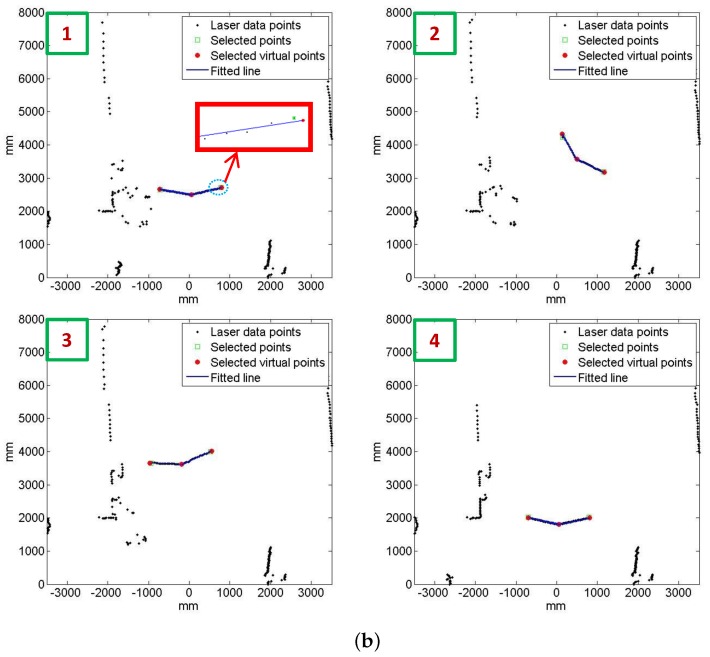
Four pairs of images and LiDAR scans are used as the ground truth. The calibration target was placed with two straight poses and two rotated poses. (**a**) Ground truth images. The blue line is the mid-line of the marked tape. The three red lines are line features extracted manually. The yellow points are the end points of each line features; (**b**) Ground truth LiDAR features. The green squares are the LiDAR points closest to both of the edges. The red dots are LiDAR features at each position. The red box shows a close-up image of the fitted line and the selected LiDAR point and the LiDAR feature of the right edge. The numbers on upper-left corner mean corresponding data between images and LiDAR data respectively.

In order to objectively evaluate our approach, we use the real LiDAR scan line on the calibration target as the ground truth. The ground truth scan line is made by marking the LiDAR scan line seen through an IR camera with colored tape as shown in Figure 5. In the evaluation, we utilize four ground truth images at distances ranging from two to eight meters. We use two objective error measures, the line alignment error and the point correspondence error. The line alignment error is the RMS distance between the LiDAR scan line points projected onto the image and the ground truth line. The point correspondence error is the residual of the projected LiDAR features with respect to the ground truth centerline and boundary positions. These evaluations are achieved using the following procedures. First, we extract the the mid-line of the marked tape and line features in the ground truth images, as shown in Figure 6a. Second, we select the corresponding point features in the LiDAR scan, as shown in Figure 6b. Third, we project the 3D points onto the ground truth image using the extrinsic parameters estimated from each algorithm. Finally, we compute the line alignment error and point correspondence error.

The effect of the calibration accuracy by the sensor noise is analyzed in a simulation environment that considers a 0.5${}^{\circ }$ resolution 2D LiDAR and 640 × 480 resolution cameras. Two cameras are placed at [0, −400, 100] and [200, −400, 100] on the LiDAR coordinate and are tilted −5${}^{\circ }$ with respect to the horizontal plane. We assume that the intrinsic parameters of both cameras are identical and have overlapping fields of view. Correspondences between the LiDAR and the cameras are obtained using a v-shaped board, and the correspondences between the cameras are acquired from the checker pattern on the v-shaped board. For the experiment, we added Gaussian noises to the image features (*σ* = 0–5 pixels) and the LiDAR data (*σ* = 0–50 mm).

## 8. Experimental Results

We evaluate the performance of the proposed extrinsic calibration approaches explained in Section 5 and Section 6 respectively.

### 8.1. Extrinsic Calibration of a LiDAR and a Camera

Our performance evaluation is four-fold; in the first experiment, we evaluated the performance of our baseline algorithm and the two extensions: the weighting method and the robust optimization. We then compared our algorithm to two state-of-the-art previously published algorithms. In the third and fourth experiments, we evaluated the effect of the number of scan/image pairs and the range and pose of the calibration target on calibration accuracy.

#### 8.1.1. Comparison of Base Line and Extension Approaches

**Figure 7:**
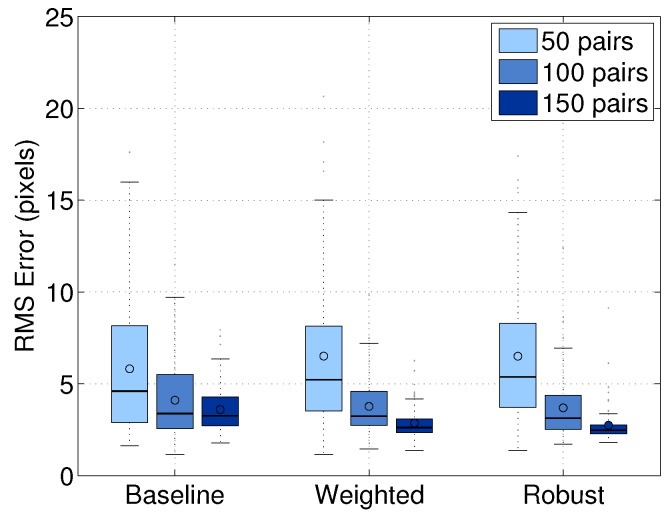
Comparison of our baseline approach with the weighted and robust extensions: the results were computed as a function of the number of image/scan pairs evaluated using 10-fold cross-validation. The errors were estimated by measuring the RMS distance with the ground truth. Circles and horizontal lines in boxes indicate the mean values and the median values of the distribution.

We compared the performance of our baseline approach and the two extensions described in Section 5. We obtain a set of 250 image/scan pairs and add noise to the 50 pairs to evaluate the robustness. We randomly selected subsets of 50, 100 and 150 image/scan pairs, in which 20% of the data are noisy. We computed the extrinsic parameters using the features extracted from each subset. The experiment was repeated multiple times using 10-fold cross-validation and using the same picked points for each image/scan pair. The results were evaluated in terms of the line alignment error averaged over the trials within each group. Figure 7 shows the comparison of our baseline, weighted and robust approaches. Within each group, the robust approach performed the best. For example, when we used 150 pairs, the robust approach performed 20% better than the baseline approach and 5% better than the weighted approach. The experiment shows that the robust approach reduces the effect of outliers in datasets. Therefore, we used the robust approach to evaluate the performance of our calibration algorithm in the following experiments.

#### 8.1.2. Comparison Against Previous Methods

**Figure 8:**
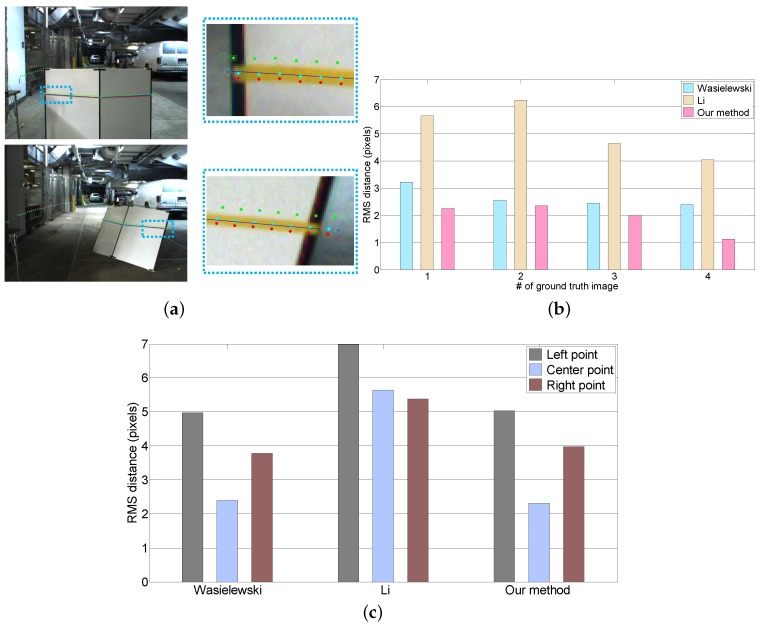
The comparison of the extrinsic calibration algorithms proposed by Walsielewski, Li and us. To do this test, we used the four ground truth images we made. (**a**) Projection of the LiDAR points onto the two ground truth images. The yellow band is the marked tape. The blue line is the mid-line of the marked tape that was manually selected. The cyan boxes show the close-up of the projection of the LiDAR points. Red points = Wasielewski; green points = Li; cyan = our method (robust); (**b**) Line alignment error between the ground truth line and projected LiDAR points; (**c**) Point correspondence error for the ground truth points and projected feature points.

We compared our method to Li’s and Wasielewski’s algorithms in terms of the error measures described in Section 7. Although Li’s approach uses a triangular calibration board and estimates the extrinsic parameters between a stereo camera and a LiDAR, we compare our approach and Li’s method because both approaches use a similar error measure. Li’s method uses two lines from the image and projected LiDAR edge points into the image coordinates. It also can be applied to the v-shaped target because the target vertical edges are not parallel in the image coordinates if the target is not perpendicular to the camera. We used the same 250 image/scan pairs as in the first experiment. Figure 8a shows the LiDAR points projected onto a test image by the extrinsic parameters acquired from each algorithm. The projected LiDAR points for our algorithm are located closer to the ground truth line than the projected points of the other methods. The performance of each algorithm is objectively evaluated by computing the line alignment error, as shown in Figure 8b. The average error of our method is 1.87 pixels. It is smaller than Wasielewski (2.7 pixels) and Li (5.5 pixels). We also evaluate the corresponding accuracy of each feature by measuring the point correspondence error (Figure 8c). In the case of Wasielewski and our method, the correspondence error of the center point is smaller than both the side points, which confirms our intuition that led to the weighted approach.

#### 8.1.3. Effect of the Number of Scan/Image Pairs

**Figure 9:**
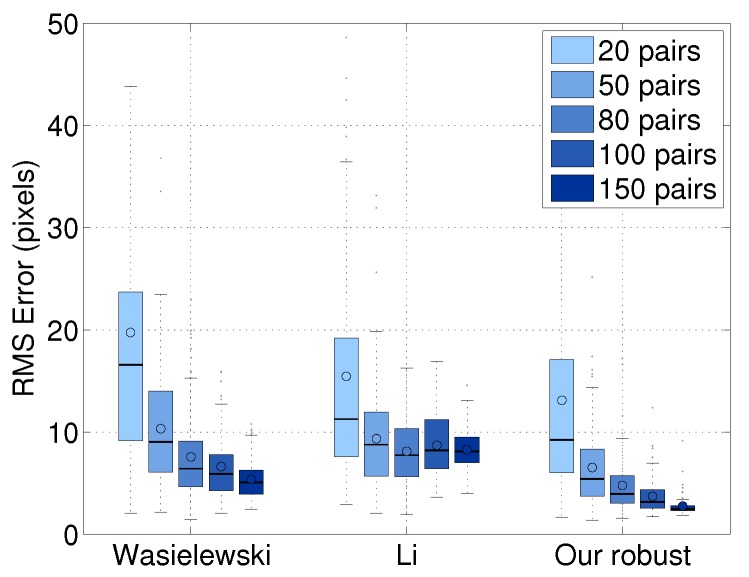
Performance as a function of number of image and LiDAR scan pairs: we used a 10-fold cross-validation to evaluate the effect that the number of calibration data has on the calibration accuracy. Our approach was also affected by the number of calibration data, but the effect was smaller than other approaches.

We evaluated the line alignment accuracy as a function of the number of image/scan pairs. This evaluation was done using 10-fold cross-validation. The number of testing image/scan pairs was randomly selected from the 250 pairs. As shown in Figure 9, our result improves when using more image/scan pairs and achieves better performance with less data. The average error of our method is 4.4 pixels. This error value is two-times lower than Wasielewski’s 8.5 pixels and 2.2-times lower than Li’s 9.6 pixels. When using 50 pairs, our approach has 43% better performance than Wasielewski’s algorithm using 100 pairs and 75% better than Li’s method with 100 pairs. This result means that our approach performs as well as the previous approaches with half of the data. It is interesting to note that the calibration accuracy of Li’s approach is not improved by increasing the number of pairs.

#### 8.1.4. Effect of the Range and Pose of the Calibration Target

**Figure 10:**
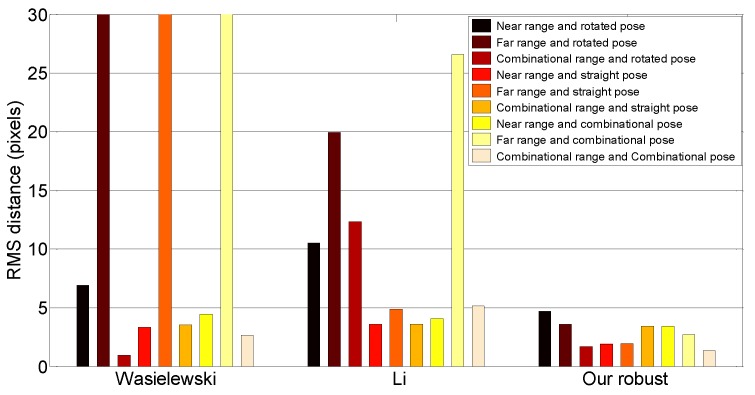
Line alignment error by the pose and range of the calibration target. Wasielewski’s method achieved over 100 pixel errors with the three far range pairs. Without these cases, Wasielewski’s method has an average of 3.75 pixel errors. The smallest line alignment error of our method is 1.32 pixels with the combination of range and poses.

We determined the effect of the pose and range of the calibration target. To do this experiment, we first labeled image/scan pairs according to the range and pose of the calibration target. The range was divided into two categories: near range (<10 m) and far range (≥10 m). We grouped the pose based on the roll angle of the target: straight (within 10${}^{\circ }$ of vertical) or rotated (more than 10${}^{\circ }$ off of vertical). Table 1 shows the number of pairs used for this experiment. Figure 10 summarizes the line alignment errors of each algorithm for each possible combination of target conditions, including combined near/far and straight/rotated groupings. Some of the methods perform poorly for specific subsets of poses. For example, Wasielewski’s method does poorly when computed using only far samples. Our method performs about the same regardless of the subset, though generally, it performs better when a combination of near/far and straight/rotated poses is used.

### 8.2. Joint Calibration of a LiDAR and Multiple Cameras

The performance of the proposed joint calibration is evaluated with synthetic and real datasets. With the synthetic data, we evaluate the calibration accuracy in the presence of the image pixel noise and the LiDAR distance noise. The real data are used to evaluate the performance of the proposed joint calibration.

#### 8.2.1. Synthetic Datasets

**Figure 11:**
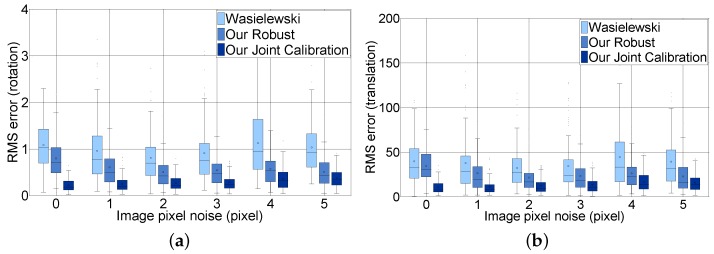
Effect of the image pixel noise using synthetic data: (**a**) RMS error of rotation in degrees and (**b**) RMS error of translation in millimeters; errors occur when the image pixel noise is zero, as our proposed algorithms uses pixel distance between the reprojected LiDAR feature and image. The error is dependent on the angular resolution and the distance to the target board.

To evaluate the effect of the image pixel noise, we compared the extrinsic calibration parameters estimated with the image noise to the ground truth that we already know. We made synthetic 100 image/scan pairs for each noise condition. We added pixel noises from 0–5 pixels with a 0.05 step in pixels and distance noises from 0–50 with one step in millimeters. For our joint calibration, we use synthetic images with fixed noise (0.15 pixels), which is usually acquired by a checkerboard detection algorithm. We estimated the extrinsic parameters in each noise condition with 100 pairs. Figure 11 shows the RMS error of rotation and translation comparing our joint calibration approach with Wasielewski’s and our robust approach without the loop closing constraints according to the image noise level for all LiDAR distance noises. The figure contains six steps from 0–5 to analyze. The experiment shows that the three methods are not sensitive to image pixel noise. Our joint calibration method outperforms the methods without loop-closing constraints in terms of the RMS error of the rotation and translation. This result arises from the fact that our joint calibration method additionally considers the errors between the images, whereas the other two methods only consider the errors between the reprojected LiDAR scan and the image pixel to solve the optimization problem. Our joint calibration method outperforms the methods without loop-closing constraints in terms of the RMS error of the rotation and translation.

**Figure 12:**
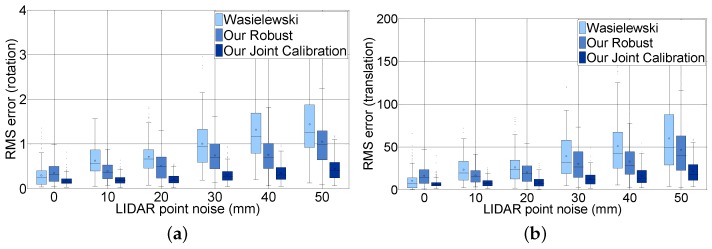
Effect of the LiDAR distance noise using synthetic data: (**a**) RMS error of rotation in degrees and (**b**) RMS error of translation in millimeters; RMS error increases proportional to the LiDAR scan noise.

Figure 12 shows the effect of LiDAR scan noise. We compare the rotation and translation error of our joint calibration with Wasielewski’s and our robust method according to the distance noise level for all image noises. The figure contains only six steps from 0–50 mm to analyze. Unlike the image pixel noise, LiDAR scan point noise has an effect on the accuracy of the extrinsic parameters. For example, when the LiDAR scan point noise increased from 0–50, the mean RMS error with respect to rotation increased from 0.18–0.44, and the mean RMS error with respect to translation increased from 7.4–21.5. The mean rotation and translation RMS error of Wasielewski’s algorithm increased five times and six times, respectively, and our robust algorithm increased three times for both rotation and translation. The accuracy of the calibration parameter with loop-closing constraints is expected to improve three times considering the fact that the sensing error of a commercial LiDAR sensor is approximately 20 mm. One can notice that the RMS error depends more on the LiDAR point noise, which can be explained by the lower resolution of the sensor.

#### 8.2.2. Real Datasets

We evaluate the performance of the joint calibration using a 3D LiDAR and two cameras mentioned in Section 7. More than 120 scan/image data pairs are used to verify the performance. The evaluation is done with N-fold cross-validation, as the ground truth for the real dataset is unknown. The robustness is demonstrated with the distribution of the calibration result from the proposed algorithm and the existing algorithm on the same randomly selected real data. Scan/image pair is a data pair of a single scan line through a v-shaped calibration target board and its corresponding imagery. The correspondences between two cameras for joint calibration are obtained from the checker pattern in near range. Because the images’ resolution is much higher than the LiDAR’s, the estimation of the relation between the two cameras is very accurate, although there is no long-range data. A sufficient amount of correspondences can be obtained from relatively less data compared to the scan/image pair.

**Figure 13:**
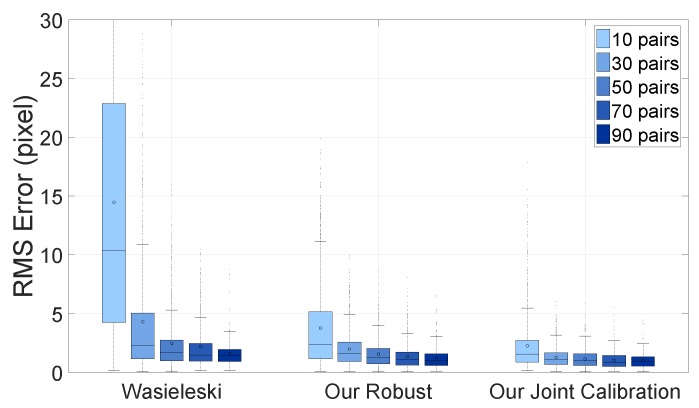
RMS distance error by the number of scan/image pairs: our joint calibration is within the 10% error bound with 30 pairs, Wasielewski with 90 pairs and our robust method with 50 pairs.

Figure 13 shows the re-projection error by the number of scan/image pairs. The N-fold cross-validation is used for this evaluation. The experiment shows that the joint calibration is more accurate than other methods given the data, and the variance of the RMS error is much smaller. In other words, the joint calibration is comparable to the single pair calibration with two- to three-times more data. For instance, joint calibration with 10 scan/image pairs has approximately 2.25 pixel errors, but Wasielewski’s method has 2.26 pixel errors with 50 pairs; and our robust method also has 2.09 pixel errors with 30 pairs. Wasielewski only uses the centerline from the v-shaped board, whereas the robust method utilizes the centerline, as well as the edge from the left and the right side of the board. The accuracy of the joint calibration is further improved by using additional camera-camera correspondence information.

**Figure 14:**
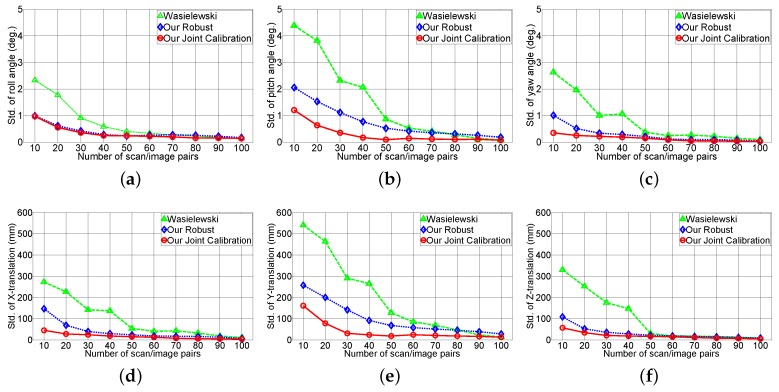
Effect of the rotation and translation accuracy w.r.t the number of scan/image pairs: (**a**–**c**) the standard deviation of the roll, pitch and yaw; and (**d**–**f**) the standard deviation of the translation on the *X*, *Y* and *Z* axes.

Figure 14 shows the effect of the rotation (roll, pith, yaw angle) and translation (*X*, *Y*, *Z* axes) accuracy with respect to the number of scan/image pairs. This experiment validates the reliability of the extrinsic parameter from the real test environment. The *X*, *Y* and *Z* axes are defined as right, down and forward, respectively, relative to the sensor. The experiment indicates our robust method is twice as robust as Wasielewski’s method given the scan/image pair, and the joint calibration method performs slightly better than the robust method. Each extrinsic parameter has different standard deviations and pitch angles. Translation along the *Y* axis has a higher standard deviation compared to the other parameters. The v-shaped calibration target is responsible for this error. Wasielewski’s method and our proposed method both use the vertical line from the v-shaped calibration board as features. The change in vertical line position is constrained with sensor the configuration because the scan line has to intersect the target’s vertical line. Therefore, variation in pitch angle and translation along the *Y* axis affect the sensor noise; whereas the roll, yaw angle and translation along *X* and *Z* axes are relatively easier to estimate with the vertical line.

## 9. Conclusions

We have presented an extrinsic calibration algorithm compensating for the drawbacks of existing approaches and providing highly accurate calibration performance. We first proposed a robust-weighted extrinsic calibration algorithm to improve the calibration accuracy between a LiDAR and a single camera. We further developed the idea and proposed the joint calibration algorithm with loop closing constraints for a sensor system with single LiDAR and two cameras, which outperforms the existing calibration algorithms. We conducted several experiments to evaluate the performance and to compare to existing state-of-the-art algorithms. The main findings of our experiments are:

- The alignment accuracy of our robust method is twice as accurate as the compared state-of-the-art algorithms.
- Our robust method requires fewer image/scan pairs to achieve the same calibration accuracy as the previous methods.
- Our method performs robustly regardless of the pose and range of the calibration target.
- With the loop-closing constraints, our joint calibration has better performance than existing state-of-the-art algorithms.

The proposed joint calibration algorithm is only feasible under a constrained sensor configuration, but the loop-closing constraint method can estimate the extrinsic parameters of the entire sensor system at once with a single objective function. If there exist overlapping regions between sensors and correspondences can be obtained, the proposed joint calibration algorithm can be applied, and more accurate parameters estimated than the existing state-of-the-art algorithms without the loop closing constraint can be obtained, as shown in the experiments.

## Figures and Tables

**Table 1:** Number of images and LiDAR scan pairs by the range and pose of the calibration target.

Range	Pose		
	Straight	Rotated	Combination
**Near**	72	146	218
**Far**	74	59	133
**Combination**	146	205	351
